# Anticancer activities of sulindac in prostate cancer cells associated with c-Jun NH2-terminal kinase 1/β-catenin signaling

**DOI:** 10.3892/ol.2014.2084

**Published:** 2014-04-25

**Authors:** JUN DU, YONGCHEN GUO, YONGHUA BAO, MENGTAO XING, ABEER M. MAHMOUD, ZHENYONG CHE, ZHIGUO CHEN, WANCAI YANG

**Affiliations:** 1 Department of Urology, Xinxiang Central Hospital, The Teaching Hospital of Xinxiang Medical University, Xinxiang, Henan 453000, P.R. China; 2 Department of Laboratory Medicine, Xinxiang Medical University, Xinxiang, Henan 453003, P.R. China; 3 Department of Microbiology, Xinxiang Medical University, Xinxiang, Henan 453003, P.R. China; 4 Department of Pathology, University of Illinois at Chicago, Chicago, IL 60612, USA; 5 Department of Neurology, Xinxiang Central Hospital, The Teaching Hospital of Xinxiang Medical University, Xinxiang, Henan 453000, P.R. China; 6 Department of Pathology, Xinxiang Medical University, Xinxiang, Henan 453003, P.R. China

**Keywords:** sulindac, prostate cancer, c-Jun NH2-terminal kinase 1, β-catenin

## Abstract

The non-steroidal anti-inflammatory agent, sulindac, has shown strong effects on cancer prevention in colorectal cancers, however, its anticancer activities on prostate cancer remain unclear. In the current study, human prostate cancer cell lines, LNCaP and PC-3, were treated with various concentrations of sulindac and it was found that sulindac significantly inhibits prostate cancer cell proliferation and promotes cell apoptosis in a dose- and time-dependent manner. Further studies revealed that sulindac significantly induces c-Jun NH2-terminal kinase (JNK) 1 phosphorylation and inhibits β-catenin at the transcriptional and post-transcriptional levels. In conclusion, by targeting the JNK1/β-catenin signaling pathway, sulindac may present a potential preventive or therapeutic agent for treatment of patients with prostate cancer.

## Introduction

Prostate cancer is one of the most common malignant diseases and the second leading cause of cancer mortality among males in the USA (1). Sulindac is a non-steroidal anti-inflammatory agent, which has shown significant activity in inhibiting gastrointestinal tumor formation in mouse models of colorectal cancers, as well as in inhibiting colorectal cancer cell proliferation and inducing cell apoptosis (2,3). However, these activities require p21, not p27 (4,5), and have been associated with c-Jun NH2-terminal kinase (JNK) 1 activation via phosphorylation *in vitro* and *in vivo* (6–8). The current study identified that sulindac exerts anticancer activities on prostate cancer cells via the inhibition of cell proliferation and induction of apoptosis by targeting the JNK1/β-catenin signaling pathway.

The JNKs have been identified as members of the mitogen-activated protein kinase family, and phosphorylate and activate various transcriptional factors, including c-Jun, activating transcription factor 2, activation protein-1 and p53 (9–13). Our previous studies demonstrated that JNK1 is critical in intestinal tumorigenesis, which was identified to be associated with p21 expression in a JNK1 gene knockout mouse model as well as colorectal cancers (14). It is also well known that the JNK signaling transduction pathway is significant in a variety of cellular processes, including cell proliferation, differentiation and apoptosis (15,16), particularly in regulating apoptosis (17–19).

To elucidate the bioactivities of sulindac and the underlying mechanism, the current study analyzed the efficacy of sulindac with regard to dosage as well as the involvement and roles of JNK1/β-catenin signaling in prostate cancer.

## Materials and methods

### Human prostate cancer cell culture

Human prostate cancer cell lines, PC-3 and LNCaP, were purchased from the American Type Culture Collection (Manassas, VA, USA) and maintained in RPMI-1640 media supplemented with 10% fetal bovine serum (FBS), 1× antibiotic/antimycotic (100 U/ml streptomycin, 100 U/ml penicillin and 0.25 μg/ml amphotericin B), 100 μM non-essential amino acids and 10 mM HEPES buffer solution (all Invitrogen Life Technologies, Carlsbad, CA, USA). All cells were cultured at 37°C in a humidified atmosphere of 5% CO_2_. Sulindac (Sigma-Aldrich, St. Louis, MO, USA) was dissolved in dimethyl sulfoxide (DMSO) and diluted in a serials concentration.

### Apoptosis analysis

The treated cells were harvested at different time points, washed in cold phosphate-buffered saline (PBS) and stained with Annexin V and propidium iodide according to the manufacturer’s instructions for the Alexa Fluor 488 Annexin V/Dead Cell Apoptosis kit (Invitrogen Life Technologies). The cells were analyzed using a CyAn ADP three channel flow cytometer and Summit3 software (both Beckman Coulter, Miami, FL, USA). The reactions were performed in triplicate and the data are representative of three independent experiments.

### Cell proliferation assay

A total of 1×10^4^ cells in 100 μl RPMI-1640 medium supplemented with 10% FBS was seeded in 96-well plates one day prior to the assay. After 18–20 h, the medium was removed and 100 μl complete assay medium was added to each well and simultaneously, sulindac was added to the medium to reach final concentrations of sulindac; 0, 0.4 and 0.8 mM. Next, 100 μl full medium with an equal volume of DMSO was added to each well as a control. All of the groups of cells were cultured in triplicate. The plates were incubated at 37°C for 24 h and the cell proliferation was determined by 3-(4,5-dimethyl thiazol-2-yl)-2,5-diphenyl tetrazolium bromide (MTT) assay (CellTiter 96 Non-Radioactive Cell Proliferation Assay kit; Promega Corporation, Madison, WI*,* USA). Briefly, 15 μl MTT (Promega Corporation) was added to each well and the plate was incubated at 7°C for 4 h in a humidified atmosphere of 5% CO_2_. Next, 100 μl stop solution was added to each well and incubated for 1 h. Finally, the absorbance was measured at 570 nm using a microplate reader (Synergy 2; BioTek Instruments, Inc., Winooski, VT, USA).

### TOP/FOP-Flash transfection and luciferase assay

To examine the effect of sulindac on β-catenin/T-cell factor (TCF) signaling, cells were seeded in 24 well-plates at a cell density of 5,000 cells/well in minimum essential media (Invitrogen Life Technologies) without antibiotics. The cells were transiently cotransfected with a TOP- or FOP-Flash plasmid (Upstate Biotechnology, Inc., Lake Placid, NY, USA) and the Renilla luciferase expression vector served as a control for transfection efficiency using Lipofectamine 2000 (Invitrogen Life Technologies). At 6 h after transfection, the medium was removed and the cells were supplied with fresh medium supplemented with 0.8 mM sulindac for 48 h and DMSO served as a control. The cells were washed with cold PBS, lysed with passive lysis buffer and luciferase activity was measured using the Dual Luciferase Report Assay System (Promega Corporation). The lysate firefly luciferase values were normalized to Renilla luciferase activity and all experiments were independently performed in triplicate.

### Immunoblotting analysis

Total protein was isolated from sulindac-treated cells, quantified by Bradford analysis and measured at 595 nm with a microplate reader using the Bio-Rad Protein Assay kit (Bio-Rad, Hercules, CA, USA). Next, 30 μg protein/lane was resolved by 10% SDS-PAGE and transferred to polyvinylidene fluoride membranes (Millipore, Bedford, MA, USA). The immunoblot was incubated overnight at 4°C with the primary antibodies, anti-JNK1 and -phosphorylated-JNK1 (p-JNK) purchased from Cell Signaling Technology, Inc. (Danvers, MA, USA). β-catenin was obtained from Sigma-Aldrich and horseradish peroxidase-conjugated affinipure goat anti-mouse IgG (Promega Corporation) secondary antibodies were used. Electrochemiluminescence western blotting detection reagents (Amersham Pharmacia Biotech, Piscataway, NJ, USA) were used as the protein signal and β-actin (Sigma-Aldrich) served as a loading control.

## Results

### Sulindac induces human prostate cancer apoptosis

**Figure 1:**
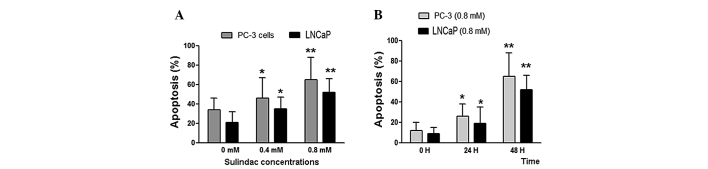
Sulindac induces human prostate cancer cell apoptosis in a dose- and time-dependent manner (human prostate cancer cell lines, LNCaP and PC-3). Cells treated with (A) 0–0.8 mM sulindac for 48 h and (B) 0.8 mM sulindac for 0–48 h were analyzed by flow cytometry. ^*^P<0.05; ^**^P<0.01 vs. the non-treatment groups.

To determine the effects of sulindac on prostate cancer cell apoptosis, PC-3 and LNCaP cells were treated with various concentrations of sulindac (0, 0.4 and 0.8 mM) for 48 h. Sulindac was found to significantly induce cell apoptosis in the two cell lines (Fig. 1A; P<0.05 at 0.4 mM and P<0.01 at 0.8 mM) compared with the untreated groups. To determine if the apoptosis was induced in a time-dependent manner, the cells were treated with 0.8 mM sulindac for 24 and 48 h. As shown in Fig. 1B, following 24 h of treatment, sulindac was found to promote apoptosis in the PC-3 and LNCaP cells (P<0.05), however, following 48 h of treatment, the induction of apoptosis was more significant (P<0.01) compared with the untreated groups. These results indicated that sulindac induces apoptosis in a dose- and time-dependent manner.

### Sulindac inhibits human prostate cancer cell proliferation

**Figure 2:**
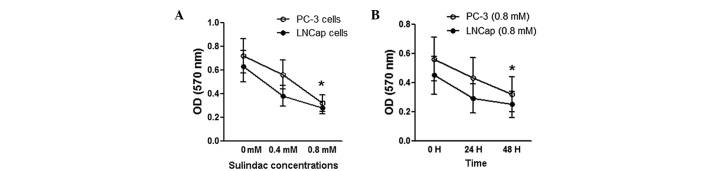
Sulindac inhibits human prostate cancer cell proliferation in a dose- and time-dependent manner (human prostate cancer cell lines, LNCaP and PC-3). Cells treated with (A) 0–0.8 mM sulindac for 48 h and (B) 0.8 mM sulindac for 0–48 h were analyzed by 3-(4,5-dimethyl thiazol-2-yl)-2,5-diphenyl tetrazolium bromide assay ^*^P<0.05 vs. the non-treatment groups. OD, optical density.

Next, the effects of sulindac on prostate cancer cell proliferation were determined. As shown in Fig. 2A, 0.8 mM sulindac was found to significantly inhibit cell proliferation in the PC-3 and LNCaP cell lines (P<0.05). However, 0.4 mM sulindac also inhibited cell proliferation in the two cell lines (P>0.05) in comparison with the untreated groups as determined by MTT. In addition, 0.8 mM sulindac was found to significantly inhibit cell proliferation following 48 h treatment (P<0.05) compared with the untreated groups (Fig. 2B). These results indicated that cell proliferation inhibition by sulindac occurred in a dose- and time-dependent manner.

### Sulindac inhibits β-catenin/TCF signaling in human prostate cancer cells

**Figure 3:**
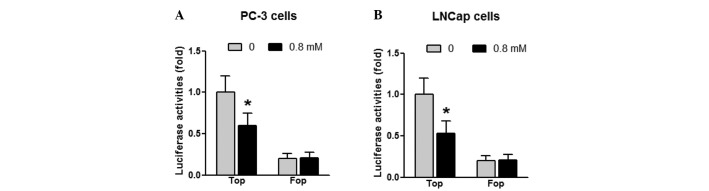
Sulindac inhibits Wnt-β-catenin at the transcriptional level as measured by analyzing the TOP/FOP-Flash luciferase activities in human prostate cancer cells. (A) PC-3 and (B) LNCaP cells cotransfected with TOP- or FOP-Flash and Renilla were treated with 0.8 mM sulindac for 48 h and harvested for luciferase analysis. FOP-Flash served as a negative control and Renilla served as an internal control. ^*^P<0.05 vs. untreated cells.

Our previous studies demonstrated that sulindac inhibits colorectal cancer cell proliferation via Wnt-β-catenin/TCF signaling (6). In the current study, it was determined that sulindac exhibits similar mechanisms within prostate cancer cells. Cells were transiently transfected with a β-catenin-TCF luciferase reporter construct, TOP-Flash, which contains multiple optimal TCF/lymphocyte enhancing factor (LEF) binding sites that induce transcription of a luciferase reporter gene when activated by β-catenin, or a negative control FOP-Flash, which contains mutant and inactivated TCF/LEF binding sites. As shown in Fig. 3, sulindac was found to inhibit β-catenin-TCF luciferase reporter activities by ~40% in PC-3 cells and ~50% in LNCaP cells (P<0.05) compared with the untreated cells.

### Sulindac suppresses β-catenin expression and induces JNK1 phosphorylation in human prostate cancer cells

**Figure 4:**
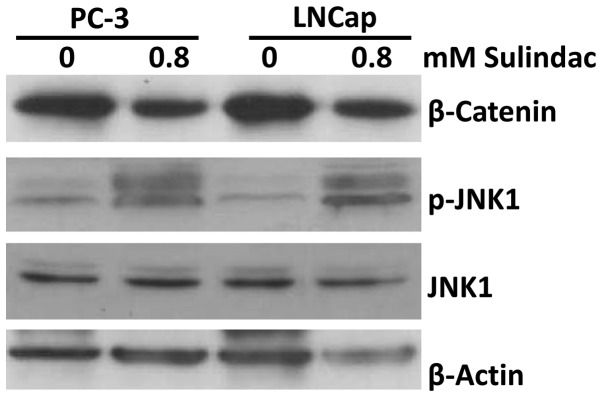
Sulindac suppresses β-catenin expression and induces JNK1 phosphorylation in human prostate cancer cells. Cells were treated with 0.8 mM sulindac for 48 h and harvested for protein extraction for immunoblotting. β-actin served as an internal control. JNK1, c-Jun NH2-terminal kinase 1.

To determine the mechanism of the sulindac-mediated induction of apoptosis and inhibition of cell proliferation in prostate cancer cells, the changes in β-catenin and JNK1 phosphorylation were investigated. Following 48 h of treatment, 0.8 mM sulindac was found to suppress β-catenin expression and induce JNK1 phosphorylation (an activated form of JNK1) in the PC-3 and LNCaP cells (Fig. 4).

## Discussion

Previous studies have shown the cancer preventive activities of sulindac on gastrointestinal cancers (4,5). The current study demonstrated that sulindac also exhibits an anticancer function within human prostate cancer cells via the promotion of cancer cell apoptosis and inhibition of cell proliferation, which was associated with the suppression of β-catenin/TCF signaling and increased JNK1 phosphorylation.

In addition to the preventive effect of sulindac on colorectal cancer, sulindac also exerts tumor inhibition on human lung and breast cancer cells (6). The present study provides evidence that sulindac influences cancer inhibition in prostate cancer cells. Similar to colorectal, lung and breast cancer, in prostate cancer cells, β-catenin is highly expressed and may present a therapeutic target for sulindac. Furthermore, sulindac was found to significantly suppress β-catenin expression at the translational and transcriptional levels, as determined by the inhibition of TOP-Flash, a vector containing multiple optimal TCF/LEF binding sites that induce transcription of a luciferase reporter gene when β-catenin is activated.

Our previous studies demonstrated that β-catenin is negatively regulated by p-JNK1 in colorectal cancers (20,21). The current study also showed that the suppression of β-catenin by sulindac is associated with increased levels of p-JNK1, although total JNK1 levels were not changed, which provides increased evidence of the involvement of sulindac in tumor inhibition by targeting the JNK1/β-catenin signaling pathway.

JNK1 has multiple functions in cell processing, particularly in response to stress, and mediates cell apoptosis and regulates cell maturation in the gastrointestinal tract (14). Based on the induction of cell apoptosis and inhibition of cell proliferation by sulindac, we hypothesize that these functions of sulindac may be associated with increased JNK1 phosphorylation and suppression of β-catenin in human prostate cancer cells.

In conclusion, sulindac exhibits anticancer activities in human prostate cancer cells by promoting apoptosis and inhibiting cell proliferation by targeting the JNK1/β-catenin signaling pathway. These findings indicate that sulindac may be a potential agent for prostate cancer prevention or therapy.

## Abbreviations

JNK1: c-Jun NH2-terminal kinase 1
